# Longitudinal quasi-static stability predicts changes in dog gait on rough terrain

**DOI:** 10.1242/jeb.149112

**Published:** 2017-05-15

**Authors:** Simon Wilshin, Michelle A. Reeve, G. Clark Haynes, Shai Revzen, Daniel E. Koditschek, Andrew J. Spence

**Affiliations:** 1 Department of Comparative Biomedical Sciences, Royal Veterinary College, London NW1 0TU, UK; 2 The National Robotics Engineering Center, Carnegie Mellon University, Pittsburgh, PA 15213, USA; 3 Department of Electrical Engineering and Computer Science, University of Michigan, Ann Arbor, MI 48109, USA; 4 Department of Electrical and Systems Engineering, University of Pennsylvania, Philadelphia, PA 19104, USA; 5 Department of Bioengineering, Temple University, Philadelphia, PA 19122, USA

**Keywords:** Quasi-static stability, Gait, Uneven terrain, Phase, Dynamical systems, Dog

## Abstract

Legged animals utilize gait selection to move effectively and must recover from environmental perturbations. We show that on rough terrain, domestic dogs, *Canis lupus familiaris*, spend more time in longitudinal quasi-statically stable patterns of movement. Here, longitudinal refers to the rostro-caudal axis. We used an existing model in the literature to quantify the longitudinal quasi-static stability of gaits neighbouring the walk, and found that trot-like gaits are more stable. We thus hypothesized that when perturbed, the rate of return to a stable gait would depend on the direction of perturbation, such that perturbations towards less quasi-statically stable patterns of movement would be more rapid than those towards more stable patterns of movement. The net result of this would be greater time spent in longitudinally quasi-statically stable patterns of movement. Limb movement patterns in which diagonal limbs were more synchronized (those more like a trot) have higher longitudinal quasi-static stability. We therefore predicted that as dogs explored possible limb configurations on rough terrain at walking speeds, the walk would shift towards trot. We gathered experimental data quantifying dog gait when perturbed by rough terrain and confirmed this prediction using GPS and inertial sensors (*n*=6, *P*<0.05). By formulating gaits as trajectories on the *n*-torus we are able to make tractable the analysis of gait similarity. These methods can be applied in a comparative study of gait control which will inform the ultimate role of the constraints and costs impacting locomotion, and have applications in diagnostic procedures for gait abnormalities, and in the development of agile legged robots.

## Abstract (precis)

**Summary:** Dogs co-ordinate their limbs on rough terrain in a manner consistent with optimization for quasi-static longitudinal stability.

## INTRODUCTION

The variability of animal movement can give insight into the ultimate costs and constraints that shape locomotion and the mechanisms that underlie it (Dickinson et al., 2000; Seyfarth et al., 2002; Westervelt et al., 2003; Diedrich and Warren, 1995; Full et al., 2002; Holmes et al., 2006; Kimura et al., 2007; Daley and Biewener, 2006; Scholz and Schöner, 1999). Here we provide evidence that variability of locomotor patterns favouring longitudinal quasi-static stability (a measure of stability defined and calculated in McGhee and Frank, 1968) can predict changes in patterns of limb co-ordination. We show how predictions based on the assumption that walking animals spend less time in longitudinally quasi-statically unstable configurations are realized in the species *Canis lupus familiaris*, the domestic dog.

While movement scientists have long considered the importance of stability in legged locomotion (Seyfarth et al., 2002; Westervelt et al., 2003; Full et al., 2002; Holmes et al., 2006; Kimura et al., 2007; Marey, 1898; Gray, 1968; Dickinson et al., 2000; Blum et al., 2007), demonstrating the influence of stability in shaping the movement of legged animals remains difficult. Ethical considerations prevent perturbing animals to the limits of stability, and even when possible, low yields are typical owing to experimental constraints [e.g. reloading a perturbation device (Jindrich and Full, 2002), or large behavioural variability]. In the face of these challenges, approaches to studying stability have included examining natural fluctuations about steady-state locomotion (Dingwell and Kang, 2007; Revzen and Guckenheimer, 2012), quantifying the recovery from significant but not catastrophic perturbations (Spence et al., 2010; Revzen et al., 2013) and considering a notion of a global stability basin outside of which the system is guaranteed or highly likely to become unstable (Dingwell and Kang, 2007). A summary of this work in humans (Bruijn et al., 2013) emphasizes the need to validate these definitions of stability, as well as the difficulty of measuring some of these quantities; it also highlights the steady progress that has been achieved in this task. For example, humans have been found to take shorter, faster and wider steps in the face of lateral perturbations when walking, in a manner that improves their margins of stability (Hak et al., 2012).

The influence of constraints and costs on animal movement is typically quantified by first describing the animal's gait (e.g. Hildebrand, 1989), and then relating a cost to that description. Often this shows that some pattern of movement (e.g. bipedal walking or running; Srinivasan and Ruina, 2005) is optimal or consistent with certain constraints. In this view, gaits are described by some prototypical, persistent pattern of movement, which we refer to as an idealized gait. An example would be the idealized trot, where diagonal pairs of limbs move exactly together. These patterns are deemed representative of the typical motion, but real animal movement is more variable: actual trots have diagonal pairs of limbs almost, but not exactly, moving in unison. Such notions of idealized gait represent a discrete concept: a sequence of steps either is a trot, or is not. Such a discrete classification precludes the notion of a pattern on a given stride being ‘more trot-like’ or ‘less trot-like’. This overlooks information that may lie in the exact gait that is utilized, when gait is considered on a continuum, rather than ‘binned’ with the nearest idealized gait, something that the pioneering work of Hildebrand (e.g. Hildebrand, 1989) noted.

One constraint that may cause animals to adjust their gait along this continuum is stability. We hypothesized that dogs would adjust their gait more rapidly if their current gait was undesirable, from the standpoint of quasi-static longitudinal stability. As a consequence, on average an animal can be predicted to spend more time in desirable stable configurations and less time in undesirable unstable ones. To test this hypothesis, we needed data in which dogs explored the neighborhood around their typical walking gait. In order to ensure dogs significantly explored the space about their steady-state walk, we trialled the dogs over rough terrain.

To detect this effect, we required methods to (1) quantify the observed gaits on a continuum, and (2) determine the longitudinal quasi-static stability of the possible gaits.

For the former task, we propose to refine the discrete idealizations of gait with an analysis based on limb phases. This analysis quantifies the gait that is exhibited on a continuum (Hildebrand, 1989), which can be thought of as a gait space. Dynamical systems approaches have had success in describing and reducing general dynamics (Kelso et al., 1986; Scholz et al., 1987; Kepler et al., 1992), as well as in the more specific context of locomotion (Diedrich and Warren, 1995; Hogan and Sternad, 2013; Aoi et al., 2013; Cohen et al., 1992). These approaches typically utilize a description that considers the phase of oscillatory components of the system. Here we apply this approach by considering each limb as an oscillator, and estimate the phase of each limb for each time point in the data set. Taking differences between these phases gives the limb phase differences, a set of numbers that is constant if the animal maintains a steady-state gait, rather than increasing with time as the individual limb phases would do. Through this analysis we can compare the dogs' gait on the flat and rough terrain.

Several approaches have been taken to quantifying the stability of observed gaits, or more broadly speaking, patterns of locomotion. For a recent comprehensive review, see Bruijn et al. (2013). Stability may be defined as the variability of a gait metric (Hausdorff, 2005), or as the time constant of some exponential recovery to a stereotyped behaviour (Dingwell and Kang, 2007). Here we adopt a pragmatic approach, utilizing a relatively simple calculation found in the robotics literature.

**Fig. 1.**
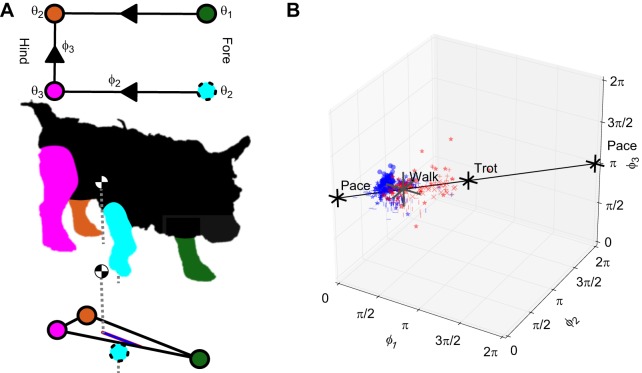
**A phase-based approach to analysis of gait incorporating considerations of quasi-static stability suggests that dogs exhibiting a gait that becomes more trot-like when walking on rough terrain is due at least in part to concern for stability.** (A) Top-down view of the limb phases and limb phase differences of a quadruped. Legs are coloured circles (hind-left limb, θ_0_, orange; fore-left, θ_1_, green; fore-right, θ_2_, cyan; rear-right, θ_3_, magenta) and arrows denote computation of phase differences, φ*_i_* (as in Eqn 1). Illustration of the longitudinal quasi-static stability margin for the case where limbs 0, 1 and 3 are on the ground. The dog is for illustrative purposes; our predictions are based on modelling, not observations of foot touch-downs. The position of the centre of mass is indicated by a black and white circle; dotted lines show the distances above the ground. The polygon of support is shown in black at the bottom of the figure; the purple line is the quasi-static longitudinal stability margin. (B) Scatter plot of gait usage by dog (371 strides, 6 subjects, marker shape as in Table 1) and terrain condition (blue, flat; red, rough), with crosses at the stereotyped walk (single-foot in lateral sequence), trot and pace. The lateral couplet walk lies to the left of walk, towards pace, approximately where the majority of blue data points are. The black line is the path connecting the single-foot walk and trot (a theoretical construct, not derived from the data).

We use the model of stability formulated by McGhee and Frank (1968), which provides a quasi-static stability measure, and that has been adapted to insect locomotion by Ting et al. (1994). In this model, the stability of a pattern of foot placements is quantified by the distance from the projection of the center of mass onto the ground to the edge of the polygon formed by the feet on the surface along the direction of motion (as depicted in Fig. 1A). The calculation measures whether the quadruped would fall over if held static in a given configuration. This is the quasi-static approximation: that the movement of the animal's limbs is treated as being sufficiently slow that it can be ignored, and only the sequence of configurations the limbs move through is taken into account.

From this analysis, we find that of the gaits neighbouring the standard walk, those in the direction of trot are most quasi-statically stable. Moving away from the idealized walk in the opposite direction, further away from trot, results in gaits that are maximally unstable. It is important to note that this analysis considers longitudinal quasi-static stability only. Trot-like patterns of motion may be desirable at walking speeds for other reasons, such as reducing pitching moment (Lee et al., 1999), or facilitation of passive stabilization (Herr and McMahon, 2000). Note that by ‘in the direction of trot’ we mean along a line in the space of limb phase differences between the idealized trot and the idealized walk.

Given that trot-like patterns have superior longitudinal quasi-static stability, we predicted that this projection would move towards zero on rough terrain in domestic dogs, indicating that the animals' walks have become more trot-like.

Our hypothesis is therefore: the relative phases of dog limbs while walking projected onto the line between walk and trot will be shifted towards trot when moving on rough terrain as compared with even terrain. The corresponding null hypothesis is: the relative phases of dog limbs while walking projected onto the line between walk and trot are consistent with no shift, or a shift away from trot, when moving on rough terrain as compared with even terrain.

We conclude by noting that while our observations are consistent with predictions based on longitudinal quasi-static stability, consideration of other alternative factors (energetics, mechanical stress, etc.) could also determine the observed motion. We discuss how this might be addressed by placing these factors in opposition, and describe subsequent experimental, comparative work that would aim to tease apart their contributions (Zelik and Kuo, 2012), and more directly predict the observed dynamics (Diedrich and Warren, 1995).

## MATERIALS AND METHODS

In overview, we are addressing two methodological problems, one theoretical and the other empirical. (1) How do we analyze kinematic data to place the exhibited gaits in a continuous space that allows distances between observed gaits to be measured? (2) How can we associate patterns of limb co-ordination with a measure of longitudinal quasi-static stability?

The answers to these questions are the central contribution of this paper and allow us to predict how dogs' limb co-ordination will change under perturbation by rough terrain. We address the first question by considering our choice of animal model system, dogs; a choice of perturbation, rough terrain; and a choice of observed variables, measurements from inertial measurement units. We then describe what we expect to see in these observations if our hypothesis about longitudinal quasi-static stability is correct, and how we process these observations to confirm this prediction.

### Animals and ethics

This study used six adult dogs of mixed sex with withers height 507.5±66.3 mm (mean±s.d.) and body mass 22.6±4.5 kg (mean±s.d.). A summary of the physical properties of the dogs (mass and withers height) is included in Table 1. We note that all of these dogs are large, none being on the scale of a Chihuahua or Basset Hound, which typically start with the more ‘trot-like’ diagonal couplet walks (Hildebrand, 1968). Each was sounded by a qualified veterinarian: none was classed as more than mildly lame in any limb. We chose dogs because they are a widely accessible animal system and rough environments on the scale of the animal's limbs are easy to find. Dogs are large enough that out of the various concerns during locomotion, quasi-static stability is likely to be important at walking speeds, and they are relatively simple to instrument non-invasively.

**Table 1.**
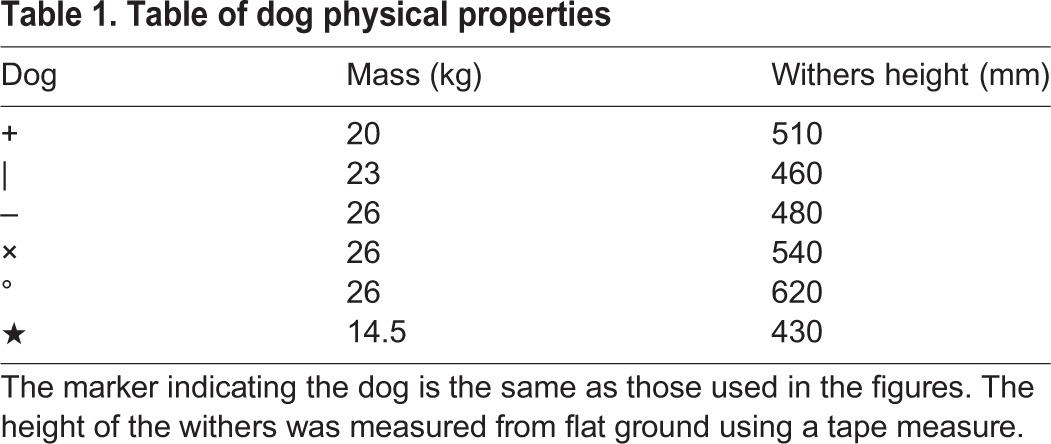
**Table of dog physical properties**

This study was approved through the Royal Veterinary College ethics review committee, number URN 2011 1091.

### Terrain

The dogs were trialled over flat and uneven terrain. A large grass sports field was used for flat terrain. Uneven terrain was a large field with longer grass, and solid, uneven ground. For the majority of the uneven terrain, perturbations were unpredictable as they were hidden by long grass. The perturbation size was measured by inserting a rod into the ground at random locations and recording the penetration distance of the stick to a reference plane. The standard deviation of these heights is a measure of the roughness of the terrain; on uneven terrain this was 54.8±44.6 mm and on flat terrain this was 4.2±3.1 mm (mean±s.d., *n*=40). The ratio of perturbation size to mean dog withers height was 0.09 on the uneven terrain and 0.006 on the flat terrain.

### Experimental protocol

Each dog was led on a leash in a straight line over flat and uneven terrain, starting at a slow speed and increasing, so that the dog progressed through walk, trot and gallop. Care was taken not to create tension in the leash.

Data collection lasted 2 days for each dog. One day began with flat terrain and then alternated rough and flat, the other began on rough terrain. For five of the six dogs, a minimum of four and a maximum of eight successful trials (runs with approximately 120 m comprising approximately equal distances of walks, trots and gallops) were completed on each terrain type within each session. For one dog, we were only able to obtain two trials on one of the uneven terrain sessions, but between four and eight successful trials for all other sessions. A successful trial was defined as one in which the dog gradually and continuously increased speed from a walk all the way through to a gallop. If the dog was distracted or suddenly changed speed within a trial, that trial was restarted.

### Data collection

Linear and angular accelerations of each leg were measured with custom-built inertial measurement units (IMUs), built by the Structure and Motion Laboratory (Royal Veterinary College), synchronized to GPS time (Tan et al., 2008). The location and speed of the dog were recorded from the GPS system.

This system contained a three-axis accelerometer (MMA7331L, Freescale Semiconductor, Tempe, AZ, USA), a single-axis gyroscope (LISY300AL, STMicroelectronics, Geneva, Switzerland) and a dual-axis gyroscope (IDG-300, InvenSense, San Jose, CA, USA). This allowed for *x*-, *y*- and *z*-axis measurements for acceleration and angular acceleration of each leg. One IMU was tightly secured to each leg using adhesive pouches constructed from Polster Plast (SnØgg Animal Polster, Strai, Kristiansand, Norway). They were positioned on the femur of the hind legs, above the stifle and below the hip, and on the humerus of the forelegs, above the elbow and below the scapula. All IMUs were placed in the same orientation. Velocity and position of the dog was measured with a custom-built GPS unit (GPS chip manufactured by u-blox, Thalwil, Switzerland, part number LEA-4T) secured onto the withers with Polster Plast. A single beacon unit containing a radio frequency communications chip was used to send a synchronization byte once per second to all four IMUs and the GPS unit. This beacon unit was attached to the dog towards the posterior of the back (loin area), again using a Polster Plast pouch.

### Data processing

Recordings from the IMUs and the GPS units were processed and synchronized using custom-written scripts in MATLAB (The MathWorks Inc., Natick, MA, USA). Regions of interest (ROIs) were selected from the raw kinematic data after low-pass filtering [third-order Butterworth (Butterworth, 1930), 5 Hz].

The ROIs chosen were whole sections of one gait, defined by constant speed movement. For all 12 combinations of dog and terrain type, in no instance was the 90th percentile of speed in the walk ROI greater than the median speed of that in trot.

On average (over the 12 combinations of dog and terrain), the median trot speed was 3.6 times the IQR of trot speed above the median walk speed. For each dog, 12 ROIs were selected with speeds characteristic of: a walk, a trot and a transverse gallop, for each of the two flat terrain sessions and the two uneven terrain sessions. Only the walks were used in this study.

These raw ROIs were then *z*-scored and low-pass filtered with a cut-off of 1 Hz using a zero phase lag digital Butterworth (Butterworth, 1930) filter from the SciPy library (http://www.scipy.org/) in a custom Python script (Python Software Foundation, Wolfeboro Falls, NH, USA). Phases of each leg were computed using the Phaser algorithm described by Revzen and Guckenheimer (2008). The Phaser method is particularly effective (compared with phase estimated from events or the Hilbert transform) for detecting small changes in timing as might be seen in a quadruped finely tuning its gait.

The zero crossing of the *z*-scored gyroscope along the axis of the hip rotation was used to define the zero phase for each leg. Phases for each leg were computed as in Fig. 1. From these phases, the phase differences (ϕ*_i_* as in Fig. 1) were calculated using Eqn 1 below. Individual strides were then cut as complete cycles of the fore left leg. Because Phaser uses the Hilbert transform in its calculation, it suffers from initial and final transients. To avoid these transients, the initial and final two strides in each ROI were removed.

Duty factor was estimated from the *z*-axis gyroscope signal. Transitions between swing and stance phases caused fast transients in the *z*-axis gyroscope signal because it was attached to the limb aligned with the sagittal plane of the animal. Foot-on events were thus estimated to occur at zero-crossings of a filtered *z*-axis gyroscope signal going positive, and foot-off events as zero-crossings going negative. The signal was processed similarly to that above (filtering, *z*-scoring) for input to Phaser, with the exception that the low-pass filter cutoff was set to 10 Hz to preserve the temporal location of fast transients within the stride.

### Phase-based analysis of limb cycling

Before we can associate a pattern of motion to a measure of quasi-static stability, we must quantify the pattern of motion itself. For a quadruped an obvious distinction between the various patterns of locomotion is the offsets between the limbs as they cycle. We quantify patterns of limb co-ordination using the phase difference between pairs of limbs. The limb phases [θ_μ_, where μ is an index indicating the leg following the conventions of Hildebrand (1989), 0 for hind-left, 1 for fore-left, 2 for fore-right and 3 for hind-right] are a measure of where in a cycle a limb is, with 0 rad corresponding to the start of a cycle and 2π rad corresponding to the end (Revzen et al., 2009). Phase differences (ϕ*_i_*, where *i* is an index ranging from 1 to 3 for the maximum three independent differences) are found by subtracting one phase from another modulo 2π:
(1)
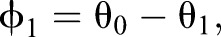

(2)
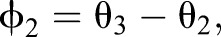

(3)
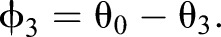


Here, ϕ_1_ and ϕ_2_ are measures of the fore–hind phase difference discussed by Hildebrand (1989) and are approximately the same for symmetric gaits. We will measure all three of these phase differences, as substantial variation in the co-ordinates would suggest that our assumptions about stability determining the phase changes of the walking gait would be incomplete.

We note that there are four limb phases and three independent phase differences; as such, Latin indices (e.g. *i*, *j*, *k*) always range from one to three, Greek indices (e.g. μ, ν) from zero to three. A phase difference quantifies how far ahead in a cycle one limb is relative to another. These limb conventions, the limb phases and the limb phase differences are depicted in Fig. 1, and are consistent with those used by Hildebrand (1989).

When four limbs move with a common frequency, which may vary over time, they will exhibit three constant independent limb phase differences (Gray et al., 1997). It is these differences that we compute to identify gait patterns. For example, in a walk, the fore-left and hind-left limbs are at a phase difference of π/2 rad. This is because at any moment in time, the hind-left is one-quarter cycle ahead of the fore-left, so hind-left phase minus fore-left phase equals positive one-quarter cycle phase difference, or π/2. Equivalently, one can think of footfall events: the hind-left foot makes contact one-quarter cycle before the fore-left. Thus ϕ_1_ is π/2. Similarly, the hind-right and fore-right limbs are at a phase difference of π/2, giving ϕ_2_. Finally, the hind-left and hind-right limbs are at a phase difference, ϕ_3_, of π. An idealized trot would have corresponding phase differences of π, π and π, with contra-lateral limbs half a cycle out of phase. Unique triples (3-tuples) of these three phase differences quantify our patterns of motion. The advantage of this approach is that it provides a compact (three numbers), yet continuous (as opposed to binned to the nearest ideal gait) quantification of gait, for each time point in the data. As such, it can quantify subtle changes in gait, how gait evolves over short time scales (e.g. within strides), and place observed gait in a ‘gait space’ relative to the ideal gaits (see Fig. 1B), or other observed gaits.

### Neighbouring gaits and trot projection

Simply computing the distance between the observed gaits and an ideal gait (e.g. the walk, at **ϕ_W_**=[ϕ_W1_,ϕ_W2_,ϕ_W3_]=[π/2,π,π/2]), with the typical equation for distance:
(4)
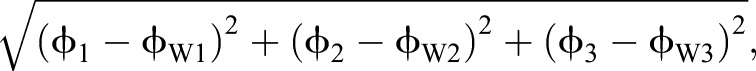


is problematic. First, distances computed in this way, in this space, would not treat deviations caused by each of the individual limb phases as equal, owing to the equations relating limb phases to limb phase differences. For example, identical changes in θ_0_ or θ_1_ would cause the resulting ϕ*_i_* to be different distances from the starting point, because θ_0_ influences two of the ϕ*_i_*, and θ_1_ only one.

This can be overcome by defining a distance metric on the individual limb phases that treats individual limbs identically, and subsequently adding a required fourth equation to the transformation between the two spaces, necessary because the individual limb phase space is four-dimensional.

Second, the observed gait points in limb phase difference space could take on a shell or donut shape in three dimensions. This would give spuriously large values of, e.g. average distance from the ideal walk, even when the central tendency of the distribution had remained at walk. To overcome this difficulty, we estimate a quantity λ, the projection of the observed gait onto the line running between walk and trot in limb phase difference space (the space having the ϕ*_i_* as co-ordinates; Fig. 1B).

More formally, in order to project the limb phase differences we have observed we must calculate an inner product on the space of limb phases. As noted above, we choose to treat all limbs as equivalent, such that differences in phase between any pair of limbs result in equal distance between gaits. This amounts to choosing a flat Euclidean metric in the space of individual limb phases (that with θ_μ_ as co-ordinates). This induces a metric on the space of φ*_i_* that we subsequently use to compute how far along the line from walk to trot a point in our space is, with our desired ‘uniform’ distance property. The resulting projection on this line implies a value of λ for each point in our space.

The squared length, *d*^2^(**ϑ**), of a vector **ϑ** with components θ_0_, θ_1_, θ_2_ and θ_3_ (corresponding to the hind-left, fore-left, fore-right and hind-right legs, respectively) in the space of limb phases is given by:
(5)
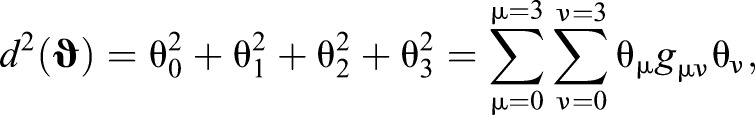
where:
(6)
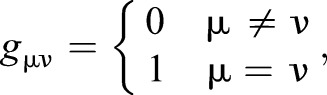


where **g** (components *g*_μν_) is a metric. Thus we treat all legs equally, and being ahead in phase for the fore-left limb (larger θ_0_) increases our distance from the origin the same amount as our hind right (larger θ_1_), and so on.

We transform co-ordinates by Eqn 1. This transformation is insufficient as we have four co-ordinates in the original space and only three in the new space. We therefore include a coordinate for a new global phase advance which is the average of the four phases. What this choice of global phase does is disregard the overall ‘mutual’ phase advance of the four limbs, leaving only information about the relative phase differences. This ‘global phase’ is defined as:
(7)
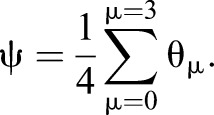


This can be written using matrices as:
(8)
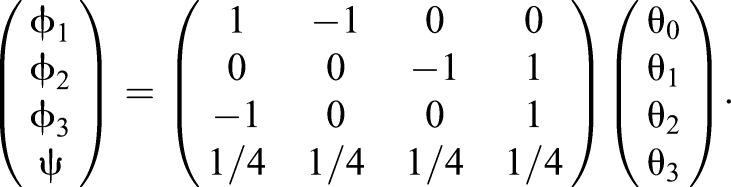


Inverting this and substituting it into Eqn 5, we find:
(9)
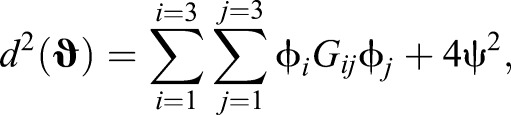
with
(10)
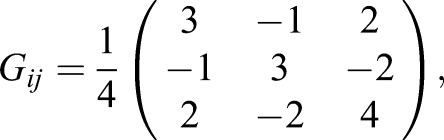
where rows correspond to *i*=1,2,3 and columns to *j*=1,2,3. This *G_ij_* is the induced metric we use to compute distances and calculate projections on the line between walk and trot (**L**_**WT**_, where the subscript W refers to walk and the subscript T to trot) in terms of the limb phase differences, ϕ*_i_*. This line is given by:
(11)
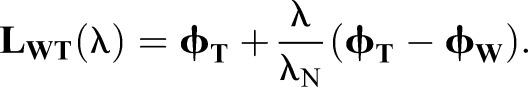
We write the components of the point **ϕ_T_** as ϕ_T0_, ϕ_T1_ and ϕ_T2_, similarly for **ϕ_W_**. If we wish to project the point **φ** (components ϕ_0_, ϕ_1_, ϕ_2_) onto this line, we calculate:
(12)
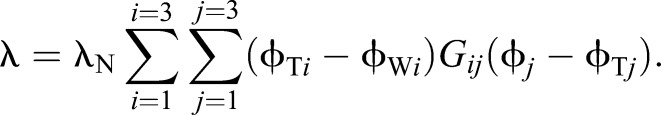


This quantity, λ, is our projection onto the line between walk and trot. The matrix **G** (components *G_ij_*) ‘does the bookkeeping’ that allows us to compute λ with the ϕ*_i_* directly, whilst ensuring that distances in this gait space depend equally on changes in phase of each limb.

The parameter λ is relatively straightforward to interpret in terms of stereotypical dog gaits. At and around λ=–π we have a pace. Between λ=–π and λ=–π/2 we have the lateral couplet walks typically associated with the larger dogs we analyze here [in Hildebrand (1968), the lateral couplet walk is defined such that λ would be between 5% and 20% of a cycle]. At and around λ=–π/2 we have the single foot walk, and between λ=–π/2 and λ=0 we have the diagonal couplet walks typically associated with smaller dogs (Hildebrand, 1968). At λ=0 we have the trot.

**Fig. 2.**
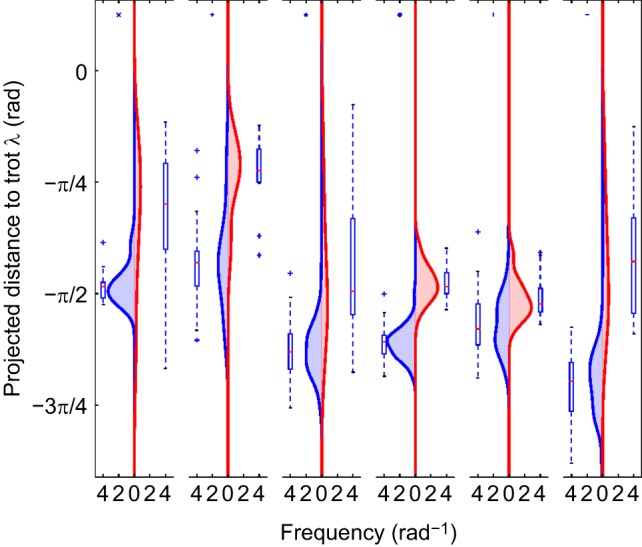
**Kernel density plots and boxplots for each dog of the values of the trot projection, λ, for flat (left; blue) and rough (right; red) terrain.** The gait is most trot-like when the projection value (λ) is zero; stereotypical (single-foot in lateral sequence) walking has a projection value of –π/2. On the rough terrain each subject is generally more trot-like. Kernel density bandwidth estimated by the Scott (2009) method implemented in the SciPy (http://www.scipy.org/) library. Here, frequency is the frequency density of strides with respect to the observed projected distance to trot. Markers in top left of each sub-plot denote the subject as in Table 1 (371 strides, 6 subjects).

We note that while the lateral couplet walk is typical for slow-moving large dogs, two of our dogs perform a single-foot walk on the flat terrain as can be seen in Fig. 2.

### Longitudinal quasi-static stability

Static stability is quantified using the longitudinal quasi-static stability margin (McGhee and Frank, 1968). The longitudinal quasi-static stability margin is the distance from the centre of mass to the front or back of the polygon of support. This model takes as input a kinematic gait formula *k* defined by:
(13)
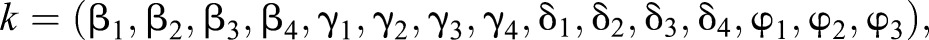
where β*_i_* is the duty factor of the *i*th leg, γ*_i_* is the fore–aft position of the *i*th leg, δ*_i_* is the vertical position of the *i*th leg and φ_i_ is the phase of the *i*th leg ahead of the first leg.

The three limb phase differences φ_2_, φ_3_ and φ_4_ are related to our three phase differences by a simple linear transformation. We will therefore simply treat *k* as a function of the phase differences in our convention. Of these three differences, there is only substantial variation along the line from walk to trot. We will therefore treat the three phase differences ϕ_1_, ϕ_2_ and ϕ_3_ as dependent only on the position on the projection on the line from walk to trot, λ. Following McGhee and Frank (1968), we will impose the restriction that all the duty cycles are identical; thus we set:
(14)
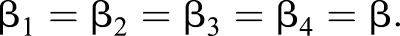


Again following McGhee and Frank (1968), we will assume that the transverse spacing between the touch-down locations of the fore-legs is identical to the spacing between the hind limbs. For real dogs, this assumption is violated, but it is necessary to make the analysis McGhee and Frank perform tractable. This condition implies:
(15)
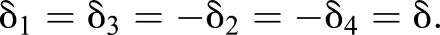


McGhee and Frank (1968) go on to show that under this assumption, the longitudinal static stability margin is insensitive to δ. We are concerned with those gaits that have high longitudinal static stability. McGhee and Frank show that those gaits where the foot touch-down locations satisfy:
(16)
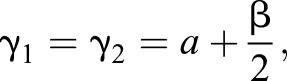


and:
(17)
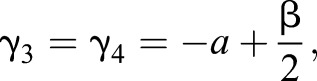
are maximally stable, where *a* is a global spacing between the fore and hind girdle. We therefore consider those gaits described by the kinematic formula given by:
(18)
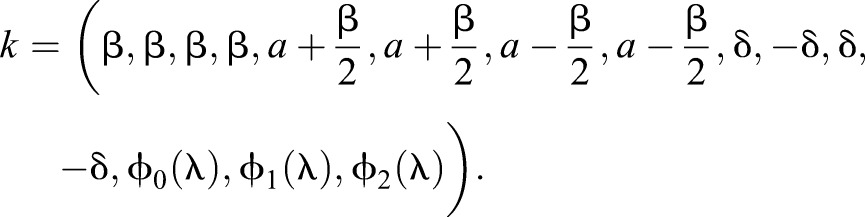


This depends only on *a*, β, δ and λ. We consider the properties of this model over the full range of observed duty factors, β. McGhee and Frank (1968) have shown that the result is insensitive to δ. We are interested in how the kinematic gait formula varies as a function of λ. This leaves *a*, a global spacing between the fore and hind girdle.

**Fig. 3.**
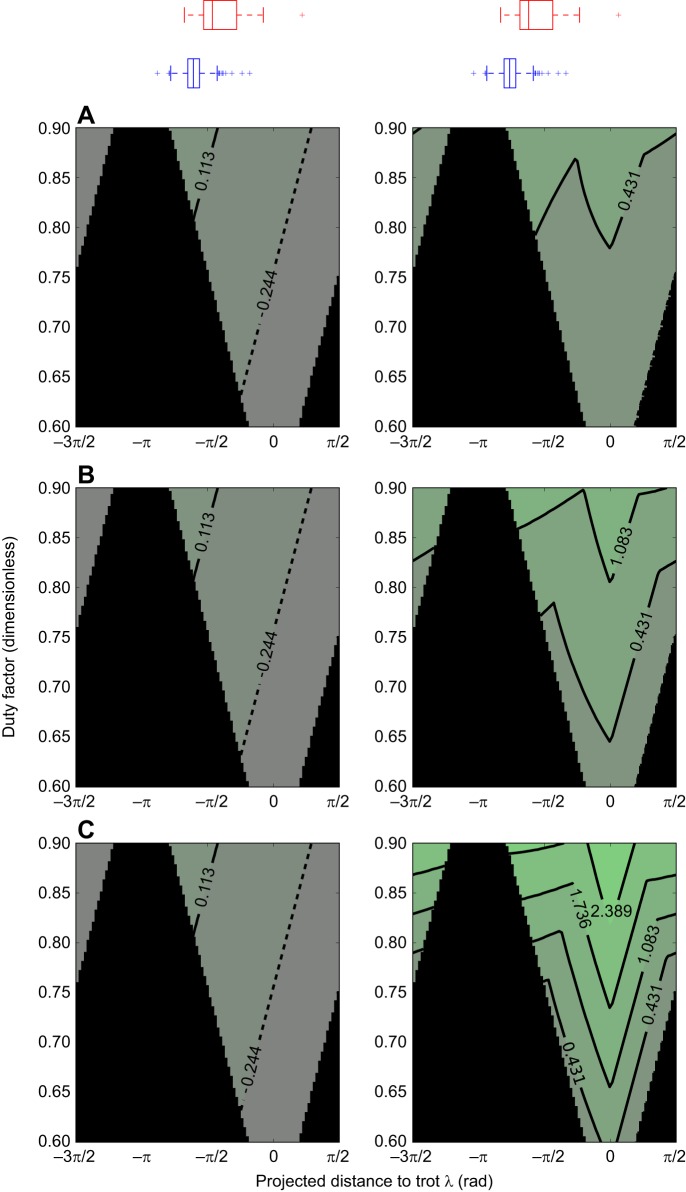
**Contour plot of longitudinal quasi-static stability margins against projected distance to trot, λ, duty factor and fore-hind touch-down spatial separation *a*, in the neighbourhood of the stereotypical (single-foot in lateral sequence) walk.** λ is varied on the *x*-axis, duty factor on the *y*-axis. Rows have different fore–hind touch-down spatial separation *a*, with (A) 0.5, (B) 1.0 and (C) 2.0. Lighter green is higher stability. The left panels display the minimum stability margin observed throughout a stride; the right panels display the average. The solid black region is where during the stride the quadruped has at least one period with no feet on the ground and the stability margin is undefined. Trot lies at zero on the *x*-axis, and the stereotypical (single-foot in lateral sequence) walk at –π/2. Irrespective of *a* and over the full range of duty factors, a shift towards trot is more desirable than away from trot. The box plots above the contour plots are of the observed λ, for all dogs (*n*=6; blue, even ground; red, uneven terrain). The gait on rough terrain shifts towards trot, where the longitudinal static stability margins are higher.

Setting δ to 1/4, we find the qualitative results are insensitive to the value of *a* (Fig. 3). Fig. 3 shows the quasi-static stability margins for *a* equal to 0.5, 1.0 and 2.0, with varying projected distance to trot λ and duty factors (from 0.6 to 0.90). We observe that across the full range of parameter values, a shift towards trot increases quasi-static stability margin when compared with a shift towards pace for single-foot or lateral couplet walks.

An intuitive grasp of the mechanism that underlies this result can be obtained by considering Fig. 9. If we begin in a lateral couplet walk and move towards trot, we move through the single-foot walk to the diagonal couplet walk. The footfall patterns of these three gaits are depicted in Fig. 9 (although we again emphasize that no evidence of discrete gait changes has been or will be presented). To obtain these plots, the duty cycle of our model was set to 0.75.

Both the lateral and diagonal couplet walks have phases that are longitudinally unstable (consistent with the results of McGhee and Frank, 1968); however, the lateral couplet walk includes a highly unstable phase where only contra-lateral limbs are in contact with the ground. These phases are far more longitudinally unstable than the corresponding phases in the lateral sequence walk, where diagonal pairs of limbs are in contact.

Although the particulars of the foot contacts change, a similar result emerges when duty factor and the aspect ratio of the animal are adjusted, as indicated in Fig. 3.

### Statistical analysis

Our statistical approach aims to ensure that the dogs are not directly switching to a trotting gait on rough terrain. This is achieved in several ways.

We confirm that the animal is travelling at walking speeds on both terrains. For all 12 combinations of dog and terrain type, in no instance was the 90th percentile of speed in the walking trials greater than the median speed of that in trotting trials.

**Fig. 4.**
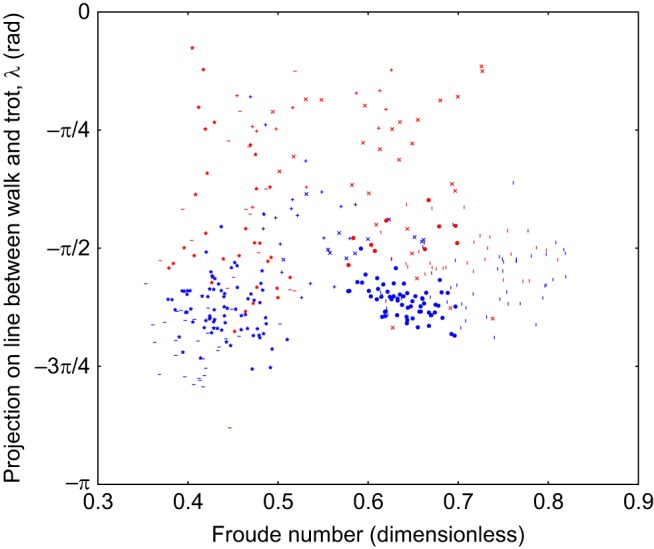
**Scatter plot of projection on the line between trot and walk against speed.** Projection is in radians, the marker shape denotes dog (371 strides, 6 subjects, as in Table 1), the colour of the points indicates terrain (blue=flat, red=rough).

**Fig. 5.**
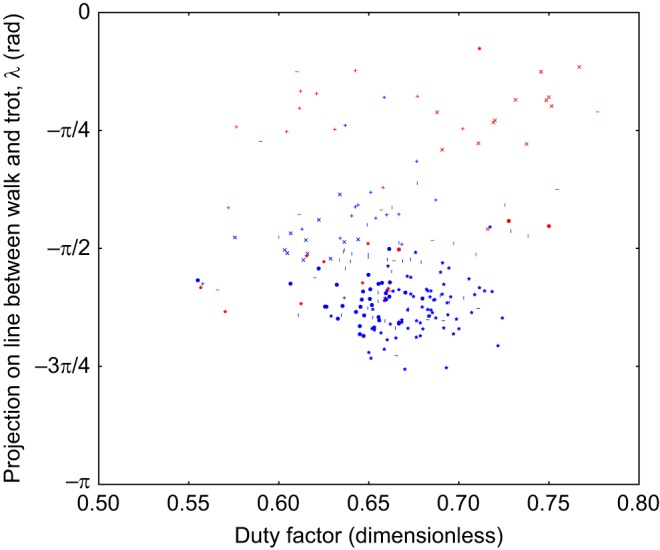
**Scatter plot of projection on the line between trot and walk against duty factor.** Projection is in radians, the marker shape denotes dog (371 strides, 6 subjects, as in Table 1), the colour of the points indicates terrain (blue=flat, red=rough). This pattern of duty factors is consistent with a shift in the limb phasing towards trot rather than a change to a trotting gait.

**Fig. 6.**
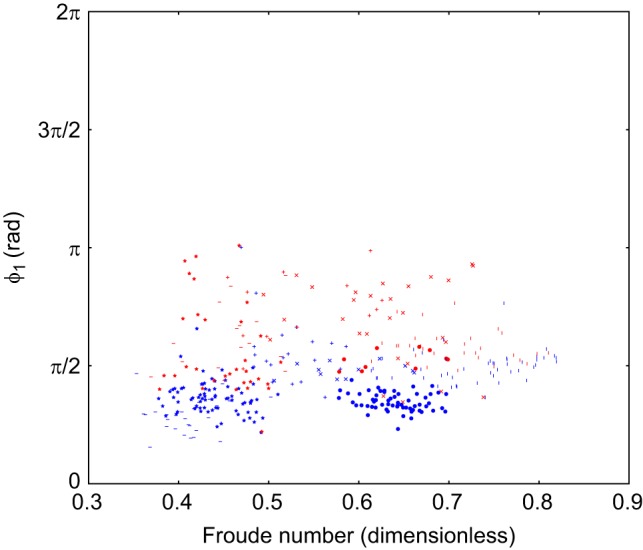
**Scatter plot of ϕ_1_ against speed.** This is the phase difference between fore-left and hind-left. Projection is in radians, the marker shape denotes dog (371 strides, 6 subjects, as in Table 1), the colour of the points indicates terrain (blue=flat, red=rough).

**Fig. 7.**
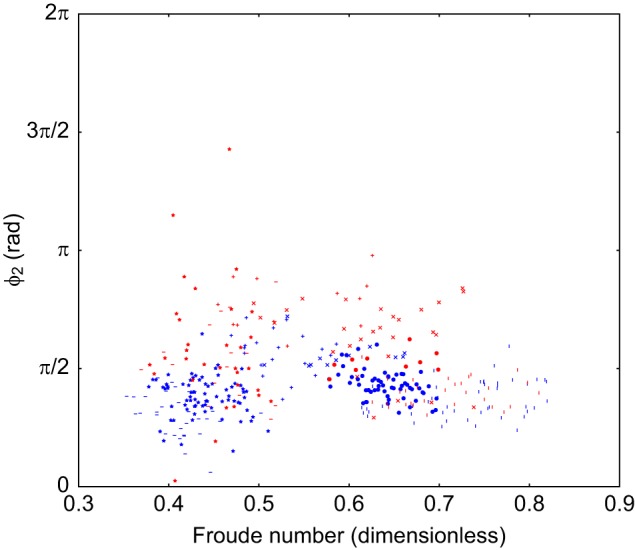
**Scatter plot of ϕ_2_ against speed.** This is the phase difference between fore-right and hind-right. Projection is in radians, the marker shape denotes dog (371 strides, 6 subjects, as in Table 1), the colour of the points indicates terrain (blue=flat, red=rough).

**Fig. 8.**
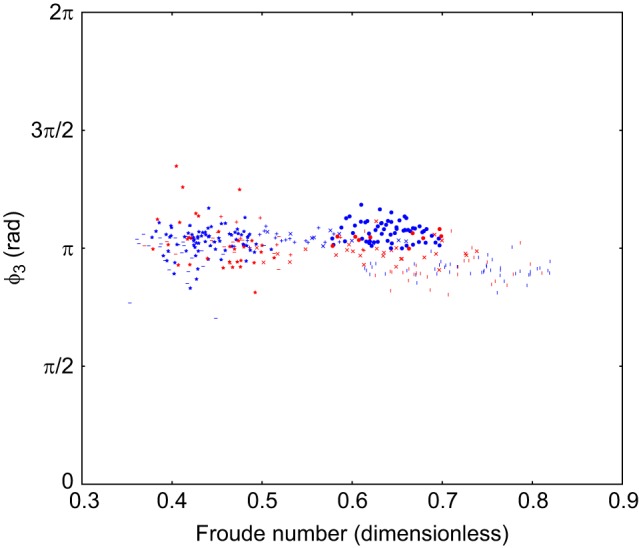
**Scatter plot of ϕ_3_ against speed.** This is the phase difference between hind-right and hind-left. Projection is in radians, the marker shape denotes dog (371 strides, 6 subjects, as in Table 1), the colour of the points indicates terrain (blue=flat, red=rough).

Scatter plots of the duty cycle, the projection along the line from trot to walk (λ) and a Froude number of the animal on a stride-by-stride basis are included in Figs 4 and 5. This Froude number is a dimensionless speed found by taking the speed of the animal and dividing it by the square root of the product of the acceleration due to gravity at the Earth's surface and the height of the withers. These show there is no relationship between the observed limb phasing and speed or duty cycle. The individual components of the observed relative phases are also included in Figs 6, 7 and 8, and indicate that there is no substantial relationship between speed and these relative phases. To further rule out this possibility, a mixed-effect model was fit with dog as a random effect, and speed and terrain type as fixed effects (R Project for Statistical Computing, http://www.r-project.org/). The error term varied by subject to compensate for heteroscedasticity and the model was fit using maximum likelihood.

## RESULTS

A total of 371 walking strides were analyzed across six subjects. This ranged between 12 and 74 strides per subject on flat terrain, and between 10 and 32 strides per subject on uneven terrain. A scatter plot of the recorded limb phasing is given in Fig. 1B. We note that there is a tight accumulation of the recorded data to the theoretical construct depicted by the black line in the figure (the straight line connecting an ideal walk to an ideal trot). The central observation of this paper is the bias of the perturbed gait data towards the trot in distinction to the expected accumulation of unperturbed gait data around the ideal walk, which is seen as the shift along this line of the red points as compared to the blue. Although the starting point along this line varied by dog, corresponding to slightly different walking gaits, for all dogs the shift from their starting point was towards trot, and hence a more stable gait.

If we take a mean of all of the trot projections obtained for each dog on the two terrain types and then perform a paired *t*-test, we confirm the prediction that at walking speeds the walking gait becomes more trot-like (*t*=–5.74, d.f.=5, *P*=0.001, mean difference is 0.531 rad). We therefore reject the null hypothesis.

A kernel density plot (and corresponding box plots) of the projections of the gait onto the circle including trot and walk is seen in Fig. 2. This highlights the shift towards trot on the rough terrain; projections of the gait on rough terrain (red) are closer to trot (λ=0) than the corresponding projections on flat terrain (blue).

While the *t*-test and kernel density plots are representative of the data and the effect observed, they do not contain corrections for differences in speed across terrain type. In the two types of terrain, the distributions of speed are comparable but not identical, and speed affects gait. We therefore confirm that the result holds in a linear mixed-effects model with speed as a covariate. We find that terrain is a significant predictor of our trot projection metric and that the sign is as expected (difference between rough and flat conditions 0.550±0.088 rad, mean±s.e.m., *t*=6.26, *P*<0.001, variance structure first-order autoregressive). Thus again we reject the null. We confirm that there is no detectable effect due to speed (gait shift with speed 0.132±0.133 rad s m^–1^, mean±s.e., *t*=1.05, *P*>0.2). The measured duty factor was 0.66±0.02 (median±IQR, *n*=6 dogs), well above that of a trotting dog.

## DISCUSSION

We tested the prediction that quadrupeds adjust their gait on rough terrain in a manner consistent with consideration for longitudinal quasi-static stability. We observe that at walking speeds, dogs exhibit a more trot-like gait on uneven terrain compared with flat terrain. All subjects showed behaviour consistent with this prediction.

While this prediction represents a success for the approach of McGhee and Frank (1968), other explanations for this observation are possible. The seeming overexploitation of trot-like patterns of motion could be viewed as supporting the work of Lee et al. (1999) and Hildebrand (1989), suggesting that trot is a stable gait choice for challenging terrain. This is also consistent with the work of Goldenberg et al. (2008), where it was found that wild black-backed jackals used a trotting gait more frequently than any other across barren sand plains, and large and small dunes with clumped vegetation.

The observed shift in limb co-ordination pattern could have a number of possible causes. We could be observing a change caused by the mechanism by which gait transitions are achieved, albeit one in which the duty cycle is not changed, and where the transition is incomplete. This could be a change from a lateral couplet walk to a single-foot walk, a single-foot walk to a diagonal couplet walk or even a (clearly unstable, non-speed dependent) transition from a walk to a trot, or some combination of the above. As noted in the Materials and methods (see Neighbouring gaits and trot projection), all of these changes constitute shifts towards trot, albeit from different starting points (the different types of walks mentioned above).

While these are possibilities, we emphasize that no discrete change in a stride parameter was observed (in the sense that might constitute a change of gait as defined by Alexander, 1984), and so inferring the presence of a new gait or a gait transition is likely premature. There may well be good reasons to suspect that the observed behaviour does not constitute a discrete change in gaits. Some of the shifts to a more trot-like limb co-ordination pattern would represent a rather odd new gait (for large dogs) between a diagonal couplet walk and a trot, assuming they are not intermittent and incomplete transitions to trot. The evidence available does not support treating this pattern of co-ordination as new discrete gait and we would urge a conservative interpretation in terms of existing known patterns of dog locomotion.

In any case, the dogs do not complete a transition to stable trot in any of the trials analysed, as can be seen in Figs 4 and 5.

**Fig. 9.**
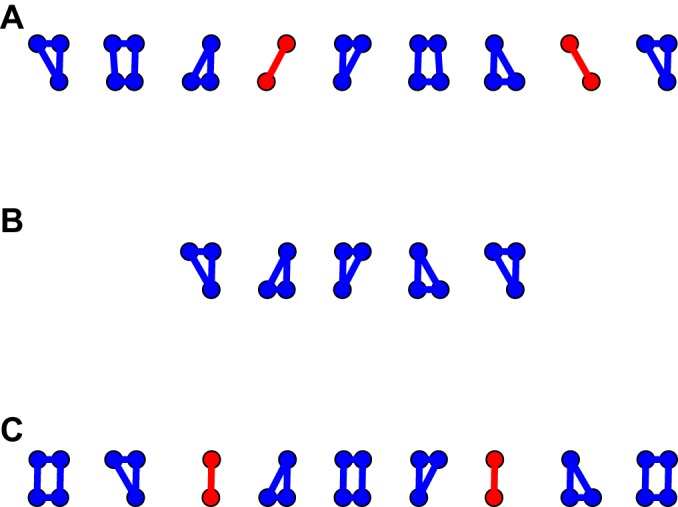
**Mechanism by which a shift towards trot enhances quasi-static stability.** Each row corresponds to footfall patterns associated with (A) a diagonal couplet walk, (B) the single-foot lateral sequence walk and (C) the lateral couplet walk (assuming a fixed duty cycle of 0.75). The single-foot lateral sequence walk is a walk in which footfalls are spaced evenly at one-quarter cycle apart, whilst the diagonal couplet walk has footfalls on ipsilateral legs more closely together, typically at 15% of a stride (and hence closer to pace, and further from trot; Hildebrand, 1968). Time evolves moving left to right across the footfall patterns, and the animal is walking from left to right across the page. The contact patterns have been separated horizontally to ease readability. As we move from bottom to top on the figure we move towards a more trot-like gait. Both the diagonal and lateral couplet walks are inferior in terms of quasi-static longitudinal stability to the single-foot walk because of the presence of the phases with only two limbs in ground contact (highlighted in red). However, for the lateral couplet walk, the pattern is especially unstable because for phases of the stride only contra-lateral pairs of limbs are in ground contact, leading to high pitch instability. For diagonal couplet walks, diagonal pairs of limbs are in contact during these unstable phases. As a result, while both these walks are longitudinally unstable, the lateral couplet walk is much more unstable.

The shifts could also be a result of the local stability of the gaits in question, some mechanically triggered mechanism, or a result of the interaction of spinal inter-neurons. The degree to which these three hypotheses are distinguishable likely rests in the ability to detect a induced gait change, which may well require a more complex perturbation.

The observed behaviour is consistent with the work in Jeka et al. (1993). It has been observed that gaits have characteristic symmetries which imply properties of the underlying dynamics (Golubitsky et al., 1999; Collins and Stewart, 1993; Schöner et al., 1990). It has been predicted and confirmed in humans moving as quadrupeds (Jeka et al., 1993) that the motion of animal limbs in the space of phase differences should be constrained to great circles. The line between walk and trot is one of these great circles, and inspection of Fig. 1B reveals that this symmetry is approximately respected by our dogs as data largely fall along this line. This suggests that this symmetry may be playing a role in shaping the response of the animal to the rough terrain perturbation.

If the observed change in limb co-ordination towards more trot-like walks is adaptive, then this work has applications in robotics via bioinspiration (Ijspeert, 2014). Further studies will implement controllers for robotic systems which replicate this gait tuning with the objective of developing robots that are more competent at traversing uneven terrain. Implementing a robotic controller that regulates phase differences is comparably simple, as robotic systems such as the RiSE family of climbing hexapods (Spenko et al., 2008) and quadrupeds (Haynes et al., 2009) in descent from their ancestral RHex family of running hexapods (Weingarten et al., 2004a; Haynes et al., 2012) and many subsequent systems are now similarly built with directly controlled limb phasing (Ijspeert et al., 2007). As such, the strategy we observe in dogs can be transferred to a diversity of robotic systems. If a robot corrects for deviations towards less quasi-statically stable patterns of locomotion at walking speeds, this should improve the quasi-static stability of the system. By measuring how much such a strategy improves (or costs) the robot, we may gather evidence to confirm or reject quasi-static stability as the ultimate driver for dogs adopting the same strategy.

A limitation of this study is the neglect of energetic cost (Hoyt and Taylor, 1981; Long and Srinivasan, 2013) and mechanical stress (Farley and Taylor, 1991), which have been shown to be predictors of gait choice and could be the determining factor for our observation of more trot-like walks on rough terrain. Indeed, past work has demonstrated changes in fore–hind limb phasing in response to altered mass distribution in dogs (Lee et al., 2004), which could act to reduce one or both of these factors. It is also possible that the perturbation is confounded by the requirement that the uneven terrain be visually obstructed by longer grass, which was not present on the flat terrain.

The effect size detected is large for three of the subjects (the first, second and last columns of Fig. 2), but is smaller for the other three. There may well be an undetected qualitative difference in strategy used by the dogs as a result of differing size, age, sex or other factors, although all six shifts are consistent with our hypothesis. With only six subjects, such an investigation is beyond the capacity of this dataset, but it seems plausible that size may be an important factor in gait selection; dogs with shorter legs have less margin in terms of time to impact if they fall, and larger or older dogs might be more concerned about stability because of the increased risk of injury from falling.

The sign of the observed (non-significant) trend between speed and λ was expected (samples at higher speed were slightly more trot-like); an increase in speed by 1 m s^−1^ would shift the gait towards trot by 0.140 rad. However, because the purpose of including this term was to allow the model to account for variations in speed confounding the observed shift to trot on rough terrain, the small impact of the term in the model suggests that speed was successfully constrained, and that while the animals’ walk became more trot-like on rough terrain, the animals were not induced to trot (as corroborated by the observed range of duty factors as observed in Fig. 5).

It remains an open question to what degree stability and energetics are decoupled; an unstable pattern of locomotion is also likely to be a costly one for animals, just as for robots (Weingarten et al., 2004b). If a concrete link between small changes in limb phasing and locomotor costs can be established, then a generalization of our approach could be used to dissect the relative contributions of these costs. Experimentally, this would require variation in intrinsic and extrinsic factors that are thought to drive gait tuning. The role of intrinsic factors could be addressed in a wider comparative study of dogs where a wide variety of morphologies and scales are available (Muybridge, 2012).

Such a concrete link could be found by expanding on the work of Zelik and Kuo (2012) and Diedrich and Warren (1995). The former have argued that by constructing locomotor costs in terms of an energy equivalent cost we can determine the relative contribution of each to the form of some locomotor task. For example, energetic cost may be measured as a rate of oxygen consumption, and stability as a distance of the centre of mass to the edge of a support polygon; by measuring the rate of oxygen consumption whilst manipulating the stability as quantified above, it would be possible to relate variation in stability with energetic cost (Donelan et al., 2004). With both of these costs expressed in the same units, their contribution may be directly compared.

The work of Diedrich and Warren (1995) shows how energetic cost can be used to predict the motion of limbs, specifically human running and walking using a dynamical equation of the form:
(19)
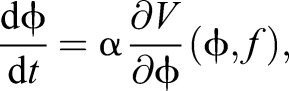
where *V* is the energetic cost of the motion, ϕ is an order parameter, *f* is some non-specific control parameter (such as cycle frequency) and α is a constant (for definitions of ‘order parameter’ and ‘non-specific control parameter’, see Revzen et al., 2009; Kelso, 1997). If stability cost can be written as *U*(ϕ,*f*) (however, note that this cannot simply be the longitudinal quasi-static stability margin because the gradient of this function contains no information about how to improve static stability in regions where the animal is maximally longitudinally unstable), then a generalized form of this equation can be written as:
(20)
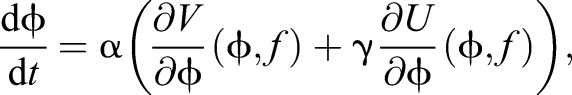
with γ the conversion factor between stability and energy analogous to that suggested by Zelik and Kuo (2012). This model can be applied to natural variability in order to tease apart the relative contribution of energetics and stability (and other factors which could be included as additional terms) to the structure of locomotion. This model, and the use of γ as a metric, would require validation as part of a larger comparative study.

### Conclusions

We have demonstrated that at walking speeds, dog walking gait shifts towards trot when terrain is uneven. Future work could examine how gait-tuning changes the metabolic cost of locomotion and forces developed in the legs, and in turn how breed, size differences and task requirements affect gait tuning.
